# The International Congress on Tuberculosis and Its Critics

**Published:** 1904-02

**Authors:** 


### TEXAS MEDICAL JOURNAL.

AUSTIN, TEXAS.

A MONTHLY JOURNAL OF MEDICINE AND SURGERY.

EDITED AND PUBLISHED BY
F. E. DANIEL, M. D.
Mrs. F. E. DANIEL, Managing Editor.

ASSOCIATE EDITOR:
WITTEN BOOTH RUSS, M. D., San Antonio, Texas.

Published Monthly at Austin, Texas. Subscription price $1.00 a year in advance
Eastern Representative: John Guy Monihan, St. Paul Building, 220 Broadway, New York City.
Official organ of the West Texas Medical Association, the Houston District Medical Association, the Austin District Medical Society, the Brazos Valley Medical Association, the Galveston County Medical Society, and several others.

### THE INTERNATIONAL CONGRESS ON TUBERCULOSIS AND ITS CRITICS.

**The Unjust and Unworthy Attack of the Editor of the Journal Amer. Med. Assn.**

Upon the suggestion and by the authority of Governor Francis and Mr. Rogers, the President and Manager of the World's Fair Exposition at St. Louis, the American Congress on Tuberculosis (organized by the Medico-Legal Society in 1900), has organized an International Congress on Tuberculosis to meet at St. Louis in October next, the committee on organization having been named by President Francis. This committee embraces some of the most distinguished physicians and sanitarians in America. The work of organization is still in progress and is nearly completed. The movement has the sanction and earnest support and co-operation of the general government, and the Department of State has invited, through our ambassadors and consuls, representation from all foreign countries. Many have responded, naming as delegates their most distinguised medical men. The French Congress on Tuberculosis, of which Professor Brouardel is President, has deferred its meeting to 1905 in order to avoid conflict with the St. Louis meeting, and that its members may unite with us at St. Louis.

Notwithstanding all this, certain medical men have bitterly attacked the movement upon the ground that it was not inaugurated by medical men alone, and certain others because they were left out or kicked out; but most surprising of all, the editor of the *Journal American Medical Association,* in a lengthy editorial (December 12, 1903), condemns and criticises the movement in a manner at once unfair, unjust and altogether unbecoming the great journal of which he has control. Moreover, he is inconsistent and contradictory. It shows prejudice towards individuals, and was evidently inspired by a letter in the same issue of the *Journal* from one Knopf, a regular wail. "Throw a stone and hear a yelp and you may know a dog is hit." I reproduce right here a copy of a letter from this Knopf, which will show the cause of his animus. It is contemptible.

NEW YORK, November 11, 1903.

"*Dr. E. J. Barrick, Toronto, Canada.*

"DEAR SIR: I have learned that there is to be an American Congress on Tuberculosis of which you are the President. I am anxious to be informed when and where the Congress is to be held, and who is the Secretary and what formalities have to be complied with to become a member? Thanking you in advance for your kindness, I am, very sincerely yours.

(Signed) "S. A. KNOPF."

"P. S.—If there is any printed matter (circulars, etc.) issued. will you kindly favor me with such?"

This letter was referred to the Executive Council of the Congress, who unanimously resolved that the connection of the applicant with the congress was not for its best interests, and the letter was never answered. Hence Knopf's wail. It was thought that Knopf was not "worth while."

There was another "great" man. His name does not exactly begin with alpha and terminate in omega, but he evidently thinks that all things begin and end in him. He was honored by the appointment to an honorary presidency, and notified of it, and the appointment was officially announced. The chairman of the committee on organization writes me as follows of this great man: "He was kicked out by the unanimous voice of the board for conduct unbecoming a gentleman. * * * He waited over four months, then knowing of the action, but secretly undermining the Congress, and did not resign or decline. He wrote a scurrilous letter, private, to a member of the Board. That letter found its way to the President, and it was regarded as so scurrilous and shameful that by unanimous action he was expelled, and his name ordered stricken off. Later, after he had published a scurrilous assault against those who had offered him a high courtesy, he sent in his resignation, and he was informed that his resignation was too late and that he had been expelled, and the official action sent him." Hence his tears.

Now, as to Editor Simmons' assault: What prompted him to say, "* * * those who are working in behalf of the organization known as the Lewis-Brown Congress are animated by pure motives,"—thus conveying the impression by inference that he thinks that those working in the interest of what he contemptuously calls the "so-called" and the "Clark Bell" Congress, are not acutated by pure motives? He thus impugns the motives and asperses the character of some of the most respected and distinguished sanitarians, lay and medical, in America. Why "so-called?" Is it not an accomplished fact? A Congress which he himself says has "the serious and earnest endorsement of the United States government," and to which already there have been appointed as delegates some of the most distinguished medical men of the world?

Dr. Simmons says that "we are neither ready nor is the time opportune for an International Congress." When it is recalled that, according to the United States census, 111,000 persons in the registration districts alone in the United States died in 1900 from consumption, it seems to me that we are "ready," or should be, if ever, to at least make an attempt at preventing its ravages; and when we recall the success with which sanitary science has dealt with other diseases—the mortality of which is insignificant compared with that of the great white plague—we should be encouraged to hope that it can also be rendered less prevalent and less fatal. Is there a single reason why an organized effort should not be made to do so? Could a more opportune time occur than during the great world's gathering at St. Louis this year, when "congresses" will constitute a large part of the program? Dr. Simmons is captious. He cavils at the action taken by the Medico-Legal Society in inaugurating this great movement for sanitary reform, and appears to be envious or chagrined because it was not done by the medical profession. Why did not the great American Medical Association move in the matter? Why did not the American Public Health Association do so? I dare say in either event Dr. Simmons would have found nothing to complain of—notwithstanding the American Public Health Association is not composed solely of physicians, but like the Congress on Tuberculosis, is made up of sanitarians from every department of science and learning, as it should be. It embraces physicians, lawyers, statesmen, legislators, engineers, educators.

The problem to be solved is not a medical one, but is medico-legal, and requires that there should be influences invoked that can secure the necessary legislation to make effective the reform that is necessary to even partly solve it; and Dr. Simmons well knows with what want of success all attempts at legislation made by the medical profession have met. In the problem of preventing consumption is embraced every feature of sanitary science, drainage, plumbing, architecture, engineering, ventilation, heating, occupation, environment. Even the killing of rats, flies, mosquitoes and fleas comes within the province of preventive medicine—sanitary science—and it embraces also all the principles of hygiene. Thus it will be seen that many of the features are not within the province of the medical practitioner.

In my deliberate opinion, Dr. Simmons has not voiced the sentiments of those whom the great *Journal of the American Medical Association* represents, and he has laid himself open to just censure for thus condemning this great movement, apparently for the reason—and only reason—that it was not instituted by the American Medical Association.

At any rate, no such flimsy objections can for a moment arrest the progress of the movement. It is as futile as the Pope's bull against the comet. The International Congress on Tuberculosis is a success: it will be held —will be largely atended by able and representative men, and it will be productive of great good, if in no other way than by awakening the people to a realizing sense of the terrible losses our people are needlessly suffering every day from a disease easily preventible, and clearly within the scope of sanitary science and legislation.

---

Surgeon General Wyman says that never again will yellow fever gain a foothold in the United States. So mote it be. It should not, and if Congress will stand by Wyman, give him all the assistance he needs, it probably will not. His powers should be enlarged. His intelligence, zeal and activity are greatly to be commended, and I, for one, think he should be at the head of a Public Health Department, and not a bureau—a sub-department within that of the treasury. The Surgeon General of the Marine Hospital and Public Health Service should be a cabinet officer, and the Surgeon General or Minister or Secretary of Public Health. He has proven his ability to fill such position with honor and credit to himself and to the whole country.

